# Durable superamphiphobic silica aerogel surfaces for the culture of 3D cellular spheroids

**DOI:** 10.1093/nsr/nwz095

**Published:** 2019-07-17

**Authors:** Lianyi Xu, Shuangshuang Chen, Xuemin Lu, Qinghua Lu

**Affiliations:** 1 School of Materials Science, Institute of Energy Equipment Materials, Shanghai Dianji University, Shanghai 201306, China; 2 School of Chemical Science and Engineering, Tongji University, Shanghai 200092, China; 3 School of Chemistry and Chemical Engineering, State Key Laboratory of Metal Matrix Composite, Shanghai Jiao Tong University, Shanghai 200240, China

**Keywords:** cell spheroids, superamphiphobicity, durability, electrochemistry, silica aerogel

## Abstract

The 3D multicellular spheroids with intact cell–cell junctions have major roles in biological research by virtue of their unique advantage of mimicking the cellular physiological environments. In this work, a durable superamphiphobic silica aerogel surface (SSAS) has been fabricated for the upward culture of 3D multicellular spheroids. Poly(3,4-ethylenedioxythiophene) (PEDOT) was first electrodeposited on a conductive steel mesh as a first template for porous silica coating. Soot particles were then applied as a second template to construct a cauliflower-like silica aerogel nanostructure. After fluorination, a hierarchical structure with re-entrant curvature was finally fabricated as a durable superamphiphobic surface. This superamphiphobic surface also presented excellent antifouling towards biomacromolecules and cells, which has been demonstrated by the successful upward culture of cell spheroids. The upward culture makes the observation of cellular behavior *in situ* possible, holding great potential for 3D cellular evaluation *in vitro*.

## INTRODUCTION

Life is 3D. The physiological environments of cells *in vivo* are inherently 3D with extensive cell–cell interactions [1]. Compared with standard 2D plasticware culture, the culture of 3D cell spheroids is considered a model for cellular physiological conditions. 3D contacts are mandatory for maintaining cellular polarity and the intracellular functions of stem cell embryos [2–4]. Furthermore, multicellular spheroids with sophisticated heterogeneous spatial distributions of oxygen and nutrition accurately mimic tumors for drug screening *in vitro* [5–7]. In this context, multicellular spheroid culture is emerging as a fundamental biological science and might provide novel insights into cancer chemotherapy. In general, 3D cellular spheroids form on non-adhesion environments where cell clusters aggregate under gravity or sheer stress. During this process, intercellular interaction molecules, especially E-cadherin, are expressed to generate compact cellular structure and inhibit the caspase-based death [8,9]. Herein, state-of-the-art strategies for multicellular spheroid culture involve the provision of an anti-adhesive bio-interface or pensile droplets for cellular self-aggregation [4,10].

Conventional methods for preparing multicellular spheroids include spinner suspension culture, hydrogel static culture, centrifugation pellet culture, floating liquid marble or magnetic nanoparticle carrying culture and hanging-droplet culture [4,6,11–15]. Most of these approaches generate heterogeneous shear stress and result in non-uniform spheroidal morphologies [16]. Hanging-drop culture methods are commonly used to culture 3D spheroids with droplets of the medium suspended from the inverted substrate surface. During culture, spheroids are efficiently formed at the bottom of the droplets with the aid of gravity and droplet curvature [17,18]. Although simple and highly efficient, the hanging-drop culture method is not suitable for *in situ* cellular spheroidal observation and characterization [19,20]. To address this difficulty, we propose an approach for upward spheroid culture for *in situ* cellular evaluation by introducing a superamphiphobic surface as a culture matrix.

Among various culture substrate materials, superhydrophobic surfaces are important bio-interface materials due to their low surface energies [21–23]. Recently, superhydrophobic surfaces have been fabricated for the generation of 3D cell spheroids by hanging-drop culture methods [6,17]. To the best of our knowledge, however, low-adhesion superamphiphobic surfaces for upward spheroid culture have not hitherto been reported. Compared with conventional hanging-drop culture, upward-culture methods based on such anti-wetting and anti-adhesion bio-interfaces permit the study of stem cell differentiation, the diffusion principle of drug delivery and tumor therapeutics *in vitro* [11,24–26]. In our earlier study, by virtue of air pockets in the micro-/nanostructures and minimization of the solid–liquid contact area, a superhydrophobic surface (Cassie’s state) was demonstrated to prevent cell adhesion [27]. Low-adhesion superhydrophobic surfaces undergo a wetting process after interacting with low-surface-tension liquids, such as various oils and surfactant solutions, leading to a loss of water repellency [28]. Superamphiphobic bio-interfaces with both superhydrophobicity and superoleophobicity are thought to be more efficient for long-term biological antifouling but are still in their infancy for real applications [29].

**Figure 1.**
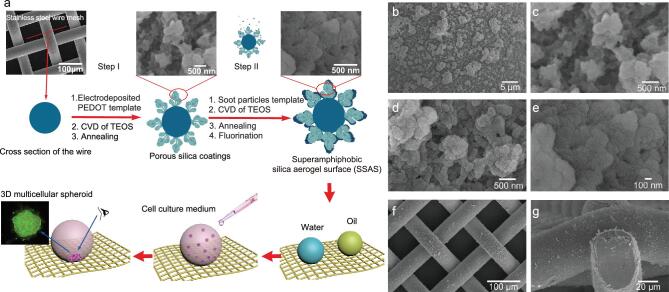
(a) Fabrication of a superamphiphobic silica aerogel surface (SSAS) by a dual-template strategy and procedure for culturing 3D cell spheroids. (b) SEM image of a porous silica coating obtained on electrodeposited PEDOT by CVD of TEOS. (c) Magnified image of the porous silica coating. (d) SEM image of the SSAS. (e) Magnified image of the SSAS. (f) SEM image of an SSAS on a stainless-steel wire mesh. (g) Lateral SEM image showing that the SSAS uniformly covered the cylindrical wires of the mesh.

In this work, we propose a novel strategy for the fabrication of a robust and durable superamphiphobic silica aerogel surface (SSAS) for multicellular spheroid culture. The SSAS was fabricated by a dual-template strategy based on electrodeposited PEDOT and soot particles as templates, coupling with the chemical vapor deposition (CVD) of tetraethoxysilane (TEOS). As presented in Fig. 1a, the electrochemical deposition of poly(3,4-ethylenedioxythiophene) (PEDOT) (as the first template) was performed on a steel mesh for the generation of a hierarchical structure. Subsequently, soot particles were introduced as a second template to enrich the hierarchical structure by further CVD. During the two templating processes, the structures were replicated by silica coating. The SSAS was finally obtained by CVD of 1*H*,1*H*,2*H*,2*H*-perfluorooctyltriethoxy- silane (POTS). The obtained SSAS displayed a cauliflower-like multiple hierarchical structure with re-entrant curvature and superamphiphobicity. It exhibited outstanding thermal and mechanical stabilities. The stable and low-adhesion SSAS was then applied for the upward culture of multicellular spheroids. Due to its excellent antifouling properties and upward-culture operation, this SSAS can be used as a universal platform for the formation of 3D cell spheroids with controlled size.

## RESULTS AND DISCUSSION

### Preparation and morphology of the SSAS

A hierarchically structured surface possessing re-entrant curvature is critically important for developing a superamphiphobic surface in the Cassie state [29]. In this work, stainless-steel wire mesh (305 mesh) was used as a conductive substrate for the electrodeposition of PEDOT (Fig. 1a, Supplementary Scheme 1, and Supplementary Fig. 1). The obtained electrodeposited PEDOT film was applied as the first template for CVD of TEOS (Supplementary Fig. 1f). After annealing at 500°C in air to remove PEDOT, the porous silica coating with hierarchical structure and re-entrant curvature was generated (Fig. 1b, c). Subsequently, in order to introduce the finer nanometer-scale structure, soot particles were deposited onto the coating as a second template (see Supplementary Scheme 2, Supplementary Figs. 2 and 3). After a second CVD of TEOS followed by annealing, the unique silica aerogel layer was finally constructed on the porous silica coating. Following fluorination by CVD of POTS, the superamphiphobic silica aerogel surface (SSAS) was successfully established (Supplementary Figs. 4–6). Figure 1c, d shows the finer nanometer-scale silica aerogel particles of size 20–30 nm uniformly covering the primary micro-/nanostructure. Compared with the porous silica coating in Fig. 1c, the SSAS was characterized by a cauliflower-like multiple hierarchical nanostructure (Fig. 1d, e). The resultant SSAS completely covered the cylindrical wires of the mesh (Fig. 1f, g). Notably, the stainless-steel wire mesh substrate also provided significant re-entrant curvature, and could promote the attainment of the Cassie state for various contacting liquid droplets. This dual-template strategy based on PEDOT film and soot particle templates provided a feasible means of preparing a superamphiphobic surface.

### Superamphiphobicity of the SSAS

**Figure 2.**
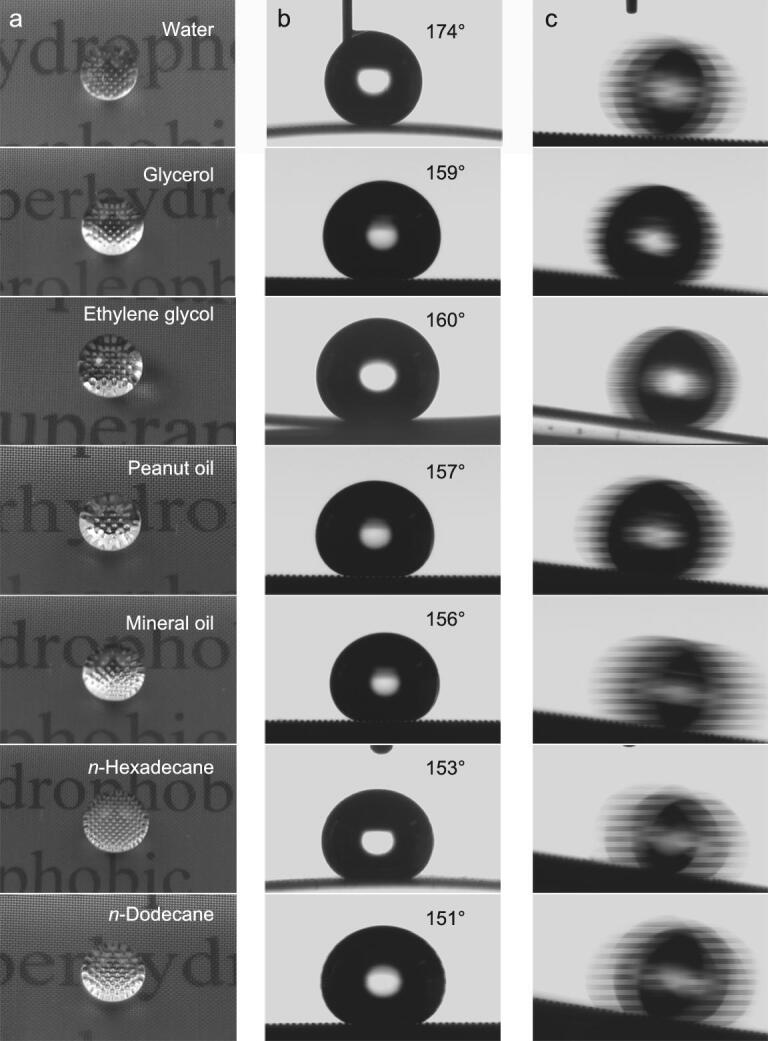
(a) Photographs of droplets of liquids with various surface tensions showing their nearly spherical shapes on the SSAS. (b) Contact angle measurements for the respective liquids on the SSAS. (c) Rolling droplets of the respective liquids showing the low sliding angles.

The cauliflower-like multiple hierarchical nanostructure coupled with low surface energy rendered the surface superamphiphobic, as evidenced by high apparent contact angles (CA or *θ* ≥ 150°) and low sliding angles (SA ≤ 10°) for liquids with various surface tensions (Fig. 2 and Supplementary Movie 1). Droplets of diverse liquids, namely water, glycerol, ethylene glycol, peanut oil, mineral oil, *n*-hexadecane and *n*-dodecane, with surface tensions ranging from 25 to 72 mN/m, were applied to evaluate the liquid repellence of the SSAS. Droplets (ca. 8 μL) of the tested liquids were randomly placed on the SSAS and acquired almost spherical shape (Fig. 2a). Detailed contact angles and sliding angles of the various liquids were quantified and are presented in Supplementary Table 1. It can clearly be seen that all contact angles exceeded 150° and that all sliding angles were less 10° (including that for *n*-dodecane with a surface tension of 25.3 mN/m). In addition, the droplets could roll away on the SSAS at very small angles, implying that the liquids could not adhere to the surface (Fig. 2c and Supplementary Movie 2). These results could be attributed to the formation of a Cassie–Baxter contact model with air pockets trapped in a hierarchical structure. The trapped air pockets would lead to liquid–solid–air composite contact interfaces and minimum solid–liquid contact areas [30,31].

**Figure 3.**
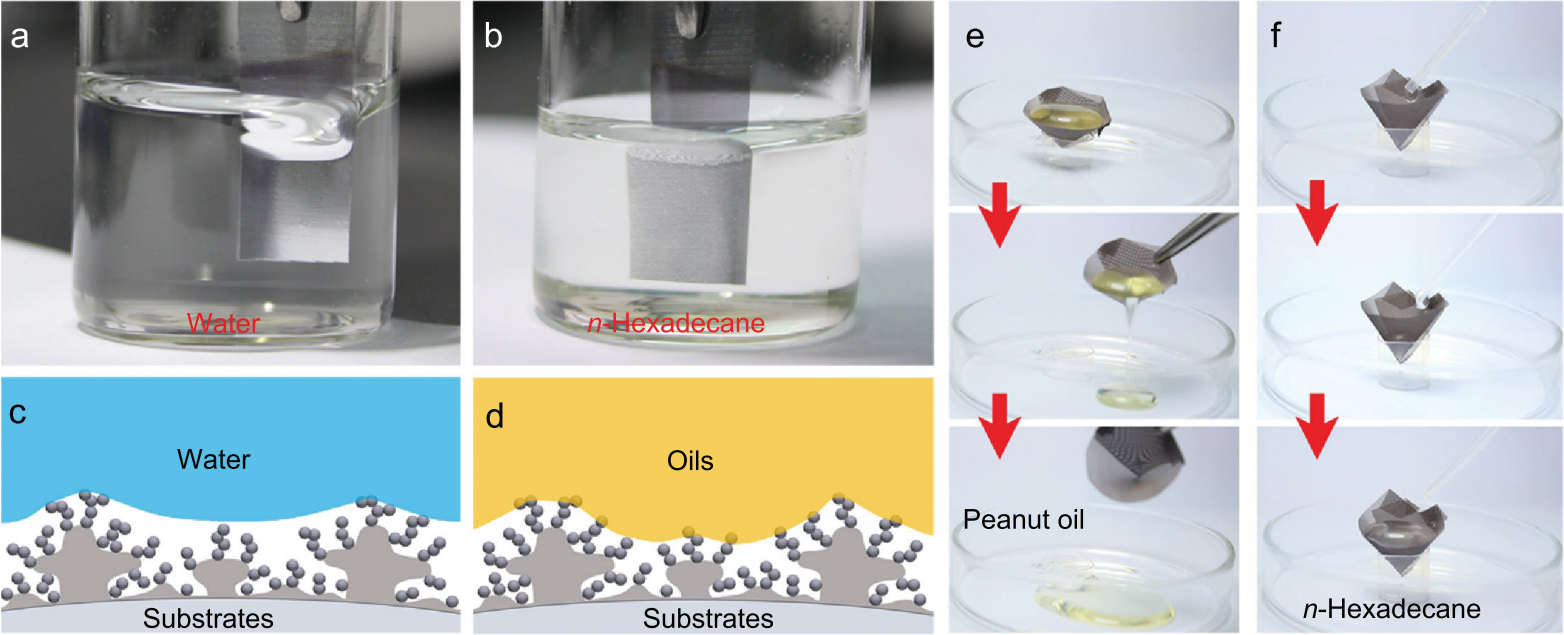
The SSAS meshes under water (a) and *n*-hexadecane (b) forming reflective interfaces like a mirror. (c, d) An illustration of an amphiphobic surface with trapped air. (e, f) SSAS can be applied to carry peanut oil and hexadecane.

The contact angle hysteresis (Δ*H*) of various liquid droplets was evaluated by measuring the advancing angle (*θ*_A_) and the receding angle (*θ*_R_) (Δ*H* = *θ*_A_ − *θ*_R_) (Supplementary Table 1 and Supplementary Fig. 7). The Δ*H* values for all liquid droplets were less than 10°, except for *n*-dodecane with the lowest surface tension (Δ*H* = 12°). The liquid repellence (superamphiphobicity) of the SSAS was further verified by immersing it in water and *n*-hexadecane. As presented in Fig. 3, the two liquids could not wet the SSAS but formed reflective mirror-like interfaces due to the trapped air pockets, indicative of a robust Cassie–Baxter state.

Both the chemical nature and physical morphology contributed to the superamphiphobicity of the SSAS. For comparison, the wettability of a fluorinated porous silica coating (FPS) obtained without the soot particle template was also evaluated in terms of apparent contact angles, sliding angles, and contact angle hysteresis (see Supplementary Fig. 8 and Supplementary Table 2). The FPS exhibited superamphiphobicity towards liquids with surface tensions higher than 27.5 mN/m, presenting CAs of

≥150°, SAs of ≤ 10° and Δ*H* values of ≤ 10°. The origin of the superamphiphobicity of the FPS could be attributed to the combination of a hierarchically structured surface with low surface energy. However, *n*-dodecane on the FPS showed only a high-adhesion pinned state with a CA of 148°. In comparison to the FPS, the SSAS showed a higher CA (151°) and a lower SA (10°) for an *n*-dodecane droplet. This result indicated that the SSAS had stronger oil repellence than the FPS, which could be attributed to the fine nanometer-scale silica aerogel particle layer, further reducing the solid–liquid contact area, thus resulting in lower solid–liquid adhesion.

### Robust and durable superamphiphobicity of the SSAS

It usually takes a few days to culture 3D multicellular spheroids. Moreover, *in situ* analyses of cellular behavior require long-term culture conditions. Hence, for real applications, the SSAS mesh needs to maintain antifouling properties for a long period. In this context, the durability of the prepared SSAS was characterized under relatively harsh conditions.

**Figure 4.**
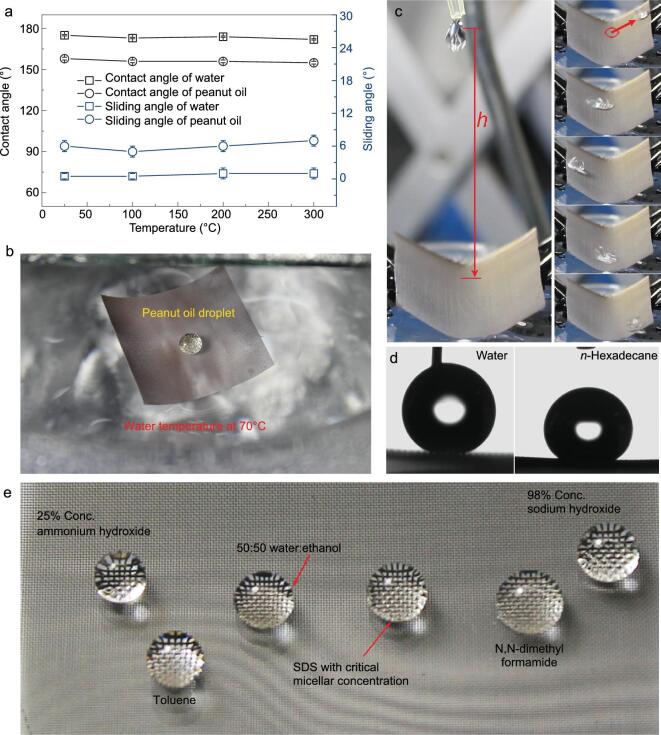
(a) Thermal stability of an SSAS mesh evaluated by measuring CA and SA of water and peanut oil after heat treatment for one hour at different temperatures. (b) Photograph of SSAS mesh with boat shape floating on hot water (70 °C) and a quasi-spherical peanut oil droplet (∼20 μL) on the SSAS mesh surface. (c) Photograph of the water droplet impact friction test devices. A water droplet (∼35 μL) was dropped from a height of ∼ 5 cm to impact onto the inside surface of the U-shaped SSAS mesh (left) and the impacting water droplets could roll back and forth several times before rolling down (right). (d) The CA measurement for water and *n*-hexadecane after 1000 drops impact friction. (e) Photograph of droplets on SSAS.

The thermal stability of the SSAS was evaluated by heating samples at different temperatures (100, 200, or 300°C) for 1 h in air. As displayed in Fig. 4a, the contact and sliding angles of water and peanut oil were well maintained after heat treatment, indicating outstanding thermal stability of the SSAS. To further study the stability of the superamphiphobicity of the SSAS under humid conditions, a sample was immersed in hot water (70°C) as shown in Fig. 4b. A boat-shaped SSAS mesh floated on the hot water (70°C) for more than 12 h. Furthermore, a peanut oil droplet (ca. 20 μL) on the SSAS mesh remained free without being pinned (see Supplementary Fig. 9 and Supplementary Movie 3). These results indicated that the SSAS mesh maintained water and oil repellence under high temperature and high humidity conditions over a long period. The stability was further evaluated under harsher conditions by immersing the SSAS mesh in boiling water for 20 min (see Supplementary Movie 4). Following this treatment, the SSAS mesh was allowed to dry naturally in air for 2 h, whereupon it retained its superamphiphobicity as demonstrated by high contact angles for water and peanut oil (164° and 151°, respectively) (Supplementary Fig. 10).

Good mechanical stability of a superamphiphobic surface is very important for real applications. In order to evaluate the mechanical properties of the SSAS, water droplet impact friction tests were performed by impacting water droplets (ca. 35 μL) from a height of 5 cm onto the inner surface of a U-shaped SSAS mesh (Fig. 4c and Supplementary Movie 5). No obvious change in the superamphiphobicity of the SSAS mesh was apparent even when it was bent into a U-shape, which could be attributed to the strong interaction between the silica coating and the substrate. After impacting 1000 drops, the SSAS exhibited high contact angles for water (165°) and *n*-hexadecane (151°) (Fig. 4d). The results indicated that the SSAS mesh had good mechanical stability.

The long-term stability of the superamphiphobicity of the SSAS was also investigated. SSAS meshes were placed in an ambient environment at about 25°C and a relative humidity of 30–60% for no less than four months. The CAs and SAs for water, peanut oil and *n*-hexadecane were then measured and showed no deviation from the original values (see Supplementary Table 3). The results indicated that the superamphiphobicity of the SSAS endured over a long period in the ambient environment.

In addition, the SSAS exhibited exceptional resistance to concentrated inorganic acid/base. High CAs and low SAs on the SSAS surface were maintained after treatment with 98% concentrated NaOH and 25% concentrated ammonia solution (Fig. 4e, Supplementary Fig. 11, and Supplementary Movie 6). The SSAS also displayed strong liquid resistance to aqueous solutions of the surfactant SDS and ethanol (50:50, H_2_O/EtOH), with CAs of 158° and 156° and SAs of  7° and 9°, respectively. Organic solvents such as toluene and DMF were also strongly repelled, with CAs in excess of 150°.

### Culture of 3D cellular spheroids on the SSAS

Various 3D multicellular spheroid culture methods have been reported. However, these previous studies did not deal with media-exchanging operations, and the methods are not suitable for long-term observation of morphological changes, leading to incomplete information. To address this, an upward spheroidal culture technique has been urgently required. The key feature of any strategy for 3D multicellular spheroid culture is to devise culture conditions that trigger cell–cell self-aggregation and prevent cell–substrate interactions [15]. As mentioned above, the prepared SSAS exhibited outstanding superamphiphobicity and good durability towards a series of liquids. Indeed, this SSAS provided a promising platform as an antifouling surface for the upward culture of 3D multicellular spheroids.

**Figure 5.**
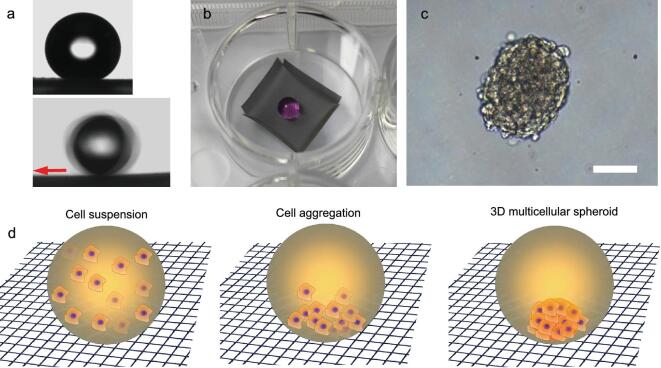
(a) The contact angle (upper) and sliding angle (lower) measurement for aqueous cell culture medium droplets (∼5 μL) on the SSAS. (b) A square container made of SSAS meshes in the well of 12-well plates and a 20-μL droplet of aqueous cell culture medium in the square container showing a quasi-spherical shape. (c) An independent compact 3D cell spheroid images after four days of culture on the SSAS surface taken using a phase contrast microscope; scale bar =100 μm. (d) The scheme of the formation of 3D cell spheroid.

As proof of concept, cell culture medium droplets (ca. 5 μL) on the SSAS displayed extremely low adhesion with a high CA of 170° and a low SA of less than 2°, indicating attainment of a Cassie–Baxter state (Fig. 5a and Supplementary Movie 7). To test the feasibility of operation, the SSAS mesh was formed into a square container without a lid (ca. 1 cm × 1 cm × 0.2 cm) to avoid droplets of the medium rolling off (Fig. 5b, Supplementary Fig. 12). The square container was then transferred to a 12-well plate (Fig. 5b). A 20 μL droplet of cell culture medium was placed in the square container; it exhibited a quasi-spherical shape and could roll freely (Fig. 5b). 20 μL of MCF-7 suspension with a cellular density of 10^5^ cells/mL was seeded in the SSAS square container. As shown in Fig. 5c, a regular 3D cell spheroid with a diameter of about 230 μm was obtained after incubation for two days. It is notable that the droplets of the cell suspension maintained a quasi-spherical shape. A suitable round bottom can be expected to improve the sedimentation of cells and enhance the formation of 3D spheroids under the gravitational force (as shown in Fig. 5d). It is interesting that the droplet containing the cell spheroids was free to roll on the SSAS mesh without being pinned (to prevent evaporation, the superamphiphobic system was surrounded by phosphate buffered solution(PBS) see Supplementary Fig. 13). In contrast, 3D cell spheroids became adhered on the surface of FPS. As mentioned above, the finer nanostructure derived from the soot particles was significant for the super-oil-phobicity. Proteins are inherently amphiphilic, and may become anchored on FPS by changing their configuration [32]. It is envisaged that cells or proteins become attached on micro-/nanometer protuberances, ultimately resulting in the formation of pinned cellular spheroids (see Supplementary Fig. 14). These results demonstrated the successful preparation of 3D multicellular spheroids on the SSAS platform by an upward-culture model. Notably, our prepared culture platform could be used multiple times to culture 3D cellular spheres without any treatment. The outstanding antifouling property and stability have been demonstrated by successfully preparing spheroids more than ten times on the same SSAS surface. Regularly cellular spheroids can be controllably and reproducibly prepared (Supplementary Fig. 15). The stable performances make SSAS a promising platform for mass production and long-term observation of spheroids.

To determine the ideal volume of culture medium droplets, we dropped liquids with a fixed cellular concentration of 10^4^ cells/mL in aliquots of 200, 100, 50, and 20 μL. The cellular aggregates after culture for two days were imaged, as shown in Supplementary Fig. 16. It can clearly be seen that the volume of culture medium played a significant role in modulating the spheroidal morphology. A larger volume led to elliptical droplets having a larger apparent flat area under their own gravity (Supplementary Fig. 16a). It is difficult for cells to self-aggregate within a relatively flat droplet bottom, leading to diffuse irregular cell aggregates. On decreasing the volume of the medium droplets, they became more spherical in shape and thus provided a curved bottom for the formation of cell spheroids. It was concluded that the optimal volume was below 50 μL. Super-low-attachment hydrogels have previously been used for *in situ* observation but more than one week was required for the formation of usable spheroids. Distinct from such super-low-adhesive hydrogel surfaces, our quasi-spherical droplets of cell suspensions on the low-adhesion SSAS allowed minimization of cell–substrate interactions and triggered cell–cell aggregation. The high efficiency endows the SSAS surface with more potentials for biological applications (illustrated by the merging of two spheroids in Supplementary Fig. 17).

**Figure 6.**
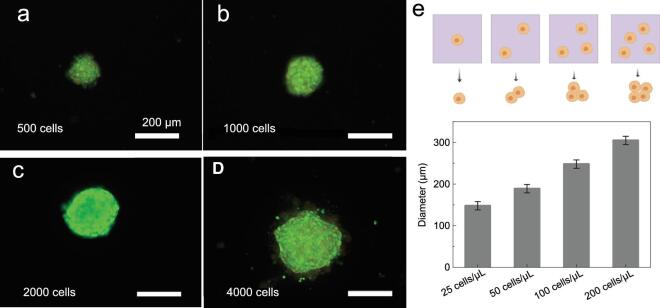
(a–d) Live/dead cell-staining images after four days of culture on the SSAS at different cell densities (25, 50, 100, or 200 cells/μL) at a fixed volume of 20 μL; scale bar = 200 μm. (e) Size distribution of multicellular spheroids with increasing cell densities from 25 to 200 cells/μL.

Spheroid size was controlled by deploying distinct numbers of cells (25, 50, 100, or 200 cells/μL) within a fixed volume of 20 μL. To further study cellular viability, the spheroids were stained with acridine orange (AO, live, corresponding to green) and ethidium bromide (EB, dead, corresponding to red) after culture for four days. As expected, the sizes of the multicellular spheroids increased with increasing cell density from 500 to 4000 cells (Fig. 6a–d). All of the prepared cell spheroids maintained a uniform spherical shape, which was significant for providing homogeneous 3D cell micromilieux for further applications. Additionally, fluorescence images verified that the viability of the spheroids was maintained on the SSAS during culture (Fig.  6e). Compared with hydration force, magnetic force and sheer stress, the intrinsic gravity was believed to be more biosafe without changing cellular physiology (Supplementary Table 4). The high viability may also be attributed to the good oxygen transportation and facile nutrition exchange by the upward-culture method. The SSAS is expected to be applicable for *in situ* analyses of drug delivery and stem embryonic development. It is notable that this method provides a common platform for various 3D cell spheroid cultures, and has successfully prepared cancerous cell spheroids (cervical carcinoma (HeLa), rat glioma cells (C6)) and non-cancerous cell spheroids (fibroblast) in Supplementary Fig. 18.

### Drug–spheroid interaction

**Figure 7.**
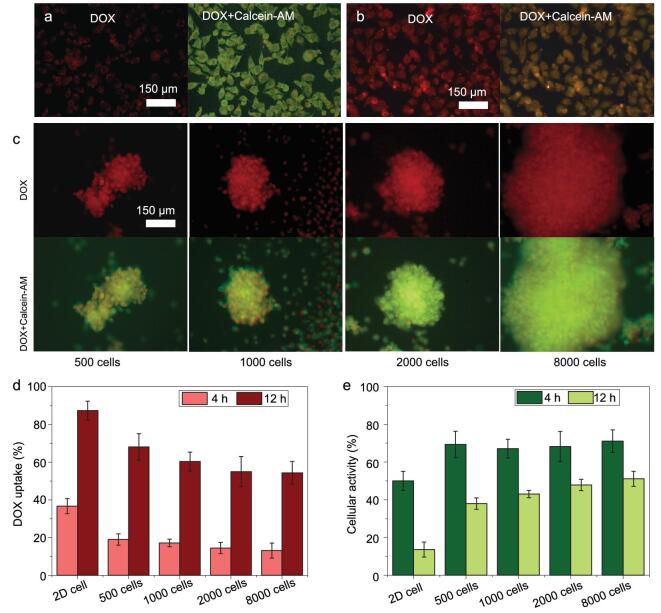
(a) Drug–DOX uptake and cell activity of a 2D cell on a polystyrene (PS) dish for 4 h. (b) DOX uptake and cell activity of a 2D cell on a polystyrene dish for 12 h. (c) DOX uptake and cell activity of 3D cell spheroids with different cell densities ranging from 500 cells to 8000 cells after drug feeding for 12 h. (d) Statistic DOX uptake of 2D cells and 3D cell spheroids. (e) Statistic cell activity of 2D cells and 3D cell spheroids.

As proof of concept, an anticancer drug was introduced after spheroid formation to study the drug–spheroid interaction. Firstly, MCF-7 spheroids of different diameters (with different cell densities ranging from 500 cells to 8000 cells) were prepared on the SSAS surface. The drug (doxorubicin, DOX) with a concentration of 30 μg/mL was added to the spheroids. Contributing to the intrinsic fluorescence of DOX (red), the drug uptake can be directly measured via fluorescent intensity. For a quantitative study, live cell indicator (Calcein-AM, green) was applied to evaluate cell activity. As shown in Fig. 7a, 2D cells exhibited a relatively good viability after DOX feeding for 4 h, but when the incubation time was extended to 12 h, most cells were dead for the quick drug uptake capacity (Fig. 7b). When it comes to 3D cell spheroids, it has been found that 3D cell spheroids exhibited slower drug uptake speeds at 4 h (Fig. 7d). With increasing culture time more drugs penetrated into 3D cellular spheroids. In this case, the drug diffusion ability was highly dependent on the spheroid sizes (Fig. 7c). The smaller spheroids exhibited better drug penetration, resulting in weaker cell activities. In addition, an active core would be found from a spheroid of 8000 cells, which might contributed to the weaker drug diffusion capacity. These results are highly consistent with drug resistance in real tumors. Herein, the SSAS offers an opportunity to construct 3D biomimetic cell conditions for tumor research.

## CONCLUSION

In conclusion, we have fabricated a robust durable superamphiphobic silica aerogel surface as a platform for the upward culture of 3D multicellular spheroids. The SSAS was fabricated on a stainless-steel wire mesh through a dual-template strategy based on electrodeposited PEDOT and soot particles as templates coupled with chemical vapor deposition. The as-fabricated SSAS displayed a cauliflower-like silica aerogel nanostructure with re-entrant curvature on both the coarse and fine scales. Abundant hierarchical structures and chemical inertness endowed the SSAS with extremely low solid–liquid adhesion for a range of liquids with different surface tensions ranging from water to *n*-dodecane. The SSAS exhibited long-term thermal and mechanical stabilities. Such a robust durable SSAS meets the requirements for long-term upward culture of 3D cell spheroids. Compact 3D cell spheroids with a consistent cell population were produced with the aid of gravity and droplet curvature in the quasi-spherical droplets of the medium. The successful formation of 3D cell spheroids of different sizes could be controlled by regulating the volume and cell density of the seeding cell suspension. Staining of the spheroids verified that their viability was maintained on the SSAS during culture. Compared with other methods, this method is efficient, biocompatible, reproducible and applicable for *in situ* observation (Supplementary Table 4). The described SSAS offers an attractive alternative to conventional hanging-drop culture methods for culturing and investigating 3D cell spheroids.

## METHODS

### Electrodeposition of the PEDOT template

Electrochemical preparation of PEDOT was performed according to our previous report (see Section 1 in the online supporting information). The working electrode was stainless-steel wire mesh (305 mesh, 3 × 6 cm^2^). Before the electrochemical process, the stainless-steel wire mesh was successively washed under ultrasonication with deionized water, absolute ethanol, and 1 M aqueous NaOH solution, and then activated by treatment with 1 M hydrochloric acid for 20 min. The activated stainless-steel wire mesh was washed with deionized water and dried in air before use. The amount of electrodeposited PEDOT film was controlled by applying an electrodeposition charge of about 180 ± 30 mC/cm^2^.

### Preparation of the hierarchically structured superamphiphobic surface

The as-prepared PEDOT film coated on stainless-steel wires was placed in a desiccator, which was closed, and then chemical vapor deposition (CVD) of tetraethoxysilane (TEOS) was performed at room temperature for 48 h according to a literature report (see Section 2 in the online supporting information) [27]. After calcining at 500°C for 2 h in air to remove the PEDOT template, a porous silica coating with a morphology similar to that of the PEDOT film was generated. The resulting coating covered each wire very well. The porous silica coating was fluorinated by performing CVD of 1*H*,1*H*,2*H*,2*H*-perfluorooctyltriethoxysilane (POTS) for 24 h.

In order to obtain a homogeneous silica aerogel layer on the as-prepared porous silica coating to further minimize the liquid–solid interaction, a feasible soot particle template method was performed according to a literature report by Deng *et al.* (see Section 3 in the online supporting information) [33]. A thin soot layer was deposited on the micro-/nanostructured surface of the aforementioned calcined porous silica coating by exposing it to a burning candle. The deposited soot particles served as a second template for generating a porous silica aerogel layer on the underlying porous silica coating. Further CVD of TEOS was performed at room temperature for 24 h with the apparatus described above. The obtained sample was subsequently calcined at 500°C for 2 h in air to remove the soot particle template and fluorinated by performing CVD of POTS for 24 h.

### Culture of 3D cell spheroids

To culture multicellular spheroids, the as-prepared SSAS mesh was shaped into small square containers without lids (1 cm × 1 cm × 0.2 cm), each of which was transferred to an individual well of a 12-well plate. Subsequently, MCF-7 cell suspension, uniformly dispersed in Dulbecco's modified eagle medium (DMEM) with 10% FBS and 1% penicillin–streptomycin at a cell concentration of about 10^5^ cells/mL, was seeded into each small square container. The cells were then incubated in an atmosphere containing 5% CO_2_ at 37°C. During the culture process, droplets of the aqueous suspension applied to the SSAS were found to be nicely spherical. After incubation, the culture medium containing cell spheroids was collected and separated. The viability of the cell spheroids was quantitatively evaluated by dead/live acridine orange/ethidium bromide (AO/EB) double staining. The 3D morphology of the cell spheroids was observed by means of an inverted fluorescence microscope (DMi8, Leica) equipped with a charge-coupled device camera.

### 3D cell spheroids for drug evaluation

MCF-7 cell spheroids with densities ranging from 500 to 8000 cells were cultured for 24 h for spheroid formation. 10 μL of DOX in PBS solution was added to each drop. After culture for 4 h and 12 h, the spheroids and 2D cells were stained with Calcein-AM according to the protocol. The stained cells were then submitted to an inverted fluorescence microscope (DMi8, Leica) equipped with a charge-coupled device camera for imaging. To quantitatively calculate the fluorescent intensity, the images were read by Software ImageJ.

## Supplementary Material

nwz095_Supplemental_Files
