# Supplementary material for: Durable superamphiphobic silica aerogel surfaces for the culture of 3D cellular spheroids
Source: Natl Sci Rev. 2019 Jul 17;6(6):1255–65. doi: 10.1093/nsr/nwz095 (PMC8291414; doi:10.1093/nsr/nwz095)
Supplement: nwz095_Supplemental_Files [file nwz095_supplemental_files.zip › Supporting_Information.docx]

**Supporting Information**

**Durable Superamphiphobic Silica Aerogel Surfaces for the Culture of 3D Cellular Spheroids**

Lianyi Xu^1,#^, Shuangshuang Chen^2,#^, Xuemin Lu^3^ and Qinghua Lu^*,2,3^.

1. School of Materials Science, Institute of Energy Equipment Materials, Shanghai Dianji University, 300 Shuihua Road, Shanghai, 201306, China

2. School of Chemical Science and Engineering, Tongji University, Shanghai, 200092, China.

3. School of Chemistry and Chemical Engineering, State Key Laboratory of Metal Matrix Composite, Shanghai Jiao Tong University, 800 Dongchuan Road, Shanghai, 200240, China

^#^ L.Y. Xu and S.S. Chen contributed equally to this work.

*Corresponding author: qhlu@sjtu.edu.cn

**Content List**

1. Electrochemical preparation of PEDOT templates

**Scheme S1**. The electrochemical cell for electrodeposition of PEDOT.

**Figure S1.** CV curve and SEM photographs of electrodeposited PEDOT.

1. Preparation of hierarchically structured porous silica coatings
2. Preparation of the superamphiphobic silica aerogel surface (SSAS)

**Scheme S2**. The preparation of the thin soot layers on porous-silica-coatings for superamphiphobic surface.

**Figure S2**. The photograph and SEM images of the thin soot layers deposited onto the porous-silica-coatings.

**Figure S3**. The SEM images of second silica aerogel surface after soot layer deposition.

**Figure S4**. XPS spectrum of the superamphiphobic silica aerogel surface (SSAS).

**Figure S5**. SEM image of the superamphiphobic silica aerogel surface (SSAS).

**Figure S6**. Cross-sectional SEM image of the superamphiphobic silica aerogel surface (SSAS).

**Figure S7**. Advancing and receding contact angles as well as sliding angles for various droplets on the SSAS surface.

**Table S1.** Contact angle (CA) and sliding angle (SA) measurements of various surface tension liquids on SSAS surface.

1. The superamphiphobicity of the fluorinated porous silica coatings (FPS)

**Figure S8.** Droplets of various surface tension liquids showing the nearly spheroid shapes on the FPS surface.

**Table S2**. Contact angle (CA) and sliding angle (SA) measurements of various surface tension liquids on FPS surface.

1. Thermal stability of the SSAS mesh

**Figure S9.** The boat made by the SSAS mesh floating on the hot water (70℃) for more than 12 hours.

**Figure S10.** Photograph of the SSAS mesh immersed into boiling water and the wettability characterization.

**Table S3.** The SSAS meshes displayed the long-team stability in a room environment.

**Table S11.** the contact angle of various liquids on SSAS surface.

1. The preparation of the small square containers.

**Figure S12.** The preparation precess of the small square containers.

1. The humidity control during culture

**Figure S13.** The illustration of humid conditions to prevent the evaporation of culture medium by placing the SSAS surface arround PBS.

1. The formation of the pinned cellular spheroids on the SPS

**Figure S14**. The image of the formation of the pinned cellular spheroids on the FPS.

1. Reproducibility

**Figure S15.** The image of multiple 3D cell spheroids and the reproducible spheroids.

1. The effect of culture medium volume on the formation of 3D cellular spheroids.

**Figure S16.** The size of droplets and the corresponding cell clusters.

1. Viability and proliferation

**Figure S17.** The optical images of cell growth and spheroid emerging after culture for 96h.

1. Cell types

**Figure S18.** The optical images of cell spheroids from cancerous Hela cells and C6 cells, and non-cancerous fibroblast cells.

1. Systematic comparison of different method for spheroid culture

**Table S4.** A systematic comparison of cell spheroid culture methods.

**Movie S1.** *n*-Hexadecane was droped continuously into the conical vessel made of SSAS mesh.

**Movie S2.** The dynamic contact of the liquids with different surface tension (including water, glycerol, ethylene glycol, peanut oil, mineral oil, *n*-hexadecane and *n*-dodecane) on SSAS.

**Movie S3.** The boat-shape SSAS mesh with peanut oil droplet (~20 μL) floating on the hot water (70℃).

**Movie S4.** SSAS mesh was immersed into boling water (100℃).

**Movie S5.** The water droplet impact friction test was performed by impacting of water droplet (~ 35μL) from a height of ~5 cm onto the inside surface of the U-shape SSAS mesh.

**Movie S6.** The rolling droplets of 25% concentrated ammonia solution, the surfactant SDS (with critical micellar concentration), ethanol (50:50 water:ethanol) and N,N-dimethylformamide on SSAS with a small inclination.

**Movie S7.** The cell culture medium droplets (~5 μL) on SSAS displayed extremely low sliding angle of less than 2°.

**Materials**

HPLC grade acetonitrile (MeCN) was provided by Shanghai Lingfeng Chemical Reagent Company. 3,4-Ethylenedioxythiophene (EDOT) (Adamas, 99%), anhydrous lithium perchlorate (LiClO_4_) (J&K, 99%), tetraethoxysilane (TEOS) (Adamas, 99%), ammonia solution (Aladdin, 25−28%), 1H,1H,2H,2H-perfluorooctyltriethoxysilane (POTS) (Alfa Aesar, 97%), n-dodecane (Adamas, 99%), n-hexadecane (Adamas, 98%), mineral oil (commercial), peanut oil (commercial), ethylene glycol (Adamas, 99%), and glycerol (Adamas, 99%) were used directly without further purification. Epithelial cancer cell line derived from breast adenocarcinoma MCF-7 cells, HeLa cells from malignant cervical tumour, glioma cell line from the nervous tumor C6 and fibroblast (C3H/10T1/2, Clone 8) were obtained from the Cell Bank of the Chinese Academy of Sciences. Culture media, including fetal bovine serum (FBS) and Dulbecco’s Modified Eagle’s Medium (DMEM) were obtained from Gibco. Other culture materials, including phosphate-buffered saline (PBS), and cell staining agents, including acridine orange/ethidium bromide (AO/EB), were purchased from Beyotime Biotechnology. The water used in this work was purified to a resistance of 18 MΩ by means of a MilliQ apparatus.

**Characterization**

Field-emission scanning electron microscopy (FE-SEM) was performed with a Nova NanoSEM instrument (FEI, USA). Static contact angle (CA) measurements in air were performed by the sessile drop method using an OCA 20 contact-angle system (Data Physics Instruments GmbH, Germany). The reported CAs are the mean values of measurements on 4 μL water droplets or 5 μL organic droplets at three different positions on each sample. Sliding angles (S_A_), advancing angles (θ_A_), and receding angles (θ_R_) were determined by slowly tilting the sample stage until the 4 μL water droplet or 5 μL organic droplet started to move. The chemical compositions of the surfaces were determined by XPS on a Kratos Axis UltraDLD spectrometer (Kratos Analytical, Ltd., Manchester, U.K.) using monochromated Al-*K*α radiation (1486.6 eV) and a take-off angle of 90°. Photographs were acquired with a camera.

**1. Electrochemical preparation of PEDOT templates**

According to our previous literature (*J. Mater. Chem. A,* 2015, 3, 3801), electrochemical preparation of PEDOT templates was performed in a three-electrode electrochemical cell by using a computer-controlled CHI 630E Electrochemical Analyzer. In this electrochemical system, stainless steel wire mesh (305 mesh, 3×6 cm^2^), platinum wire (1 mm diameter), and Ag/AgCl wire were used as working, counter, and quasi-reference electrode, respectively (Scheme S1). Before the electrochemical experiment, stainless steel wire mesh was washed successively under ultrasonication with deionized water, absolute ethanol and 1M NaOH solution, and then activated 20 min in 1M hydrochloric acid. The activated stainless steel wire mesh was washed with deionized water, dried in air before use. The electrochemical experiments were performed at room temperature and less than 40% relative humidity. PEDOT films as hierarchically structured template was electrodeposited onto stainless steel wire mesh electrodes by using cyclic voltammetry (CV) between −0.7 V and +1.6 V in 0.01 M EDOT/ACN solution containing 0.2 M LiClO_4_ as a supporting electrolyte (Figure S1a). The amount of electrodeposited PEDOT was controlled with electrodeposition charge of 180±30 mC/cm^2^. The prepared PEDOT films were rinsed three times with ethanol (95%) and dried under an air stream at room temperature. As presented in Figure S1b, the PEDOT films was coated around the wires very well (Figure S1b). Figure S1c~S1e were the scanning electron microscope (SEM) images of the PEDOT film and the film have the highly porous network-like nanostructures with some protuberances.


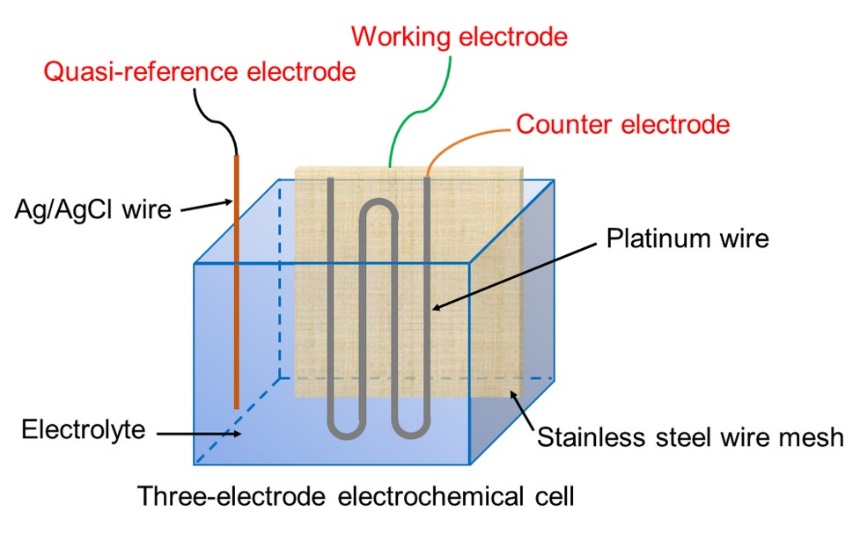


**Scheme S1.** The electrochemical cell for electrodepostion of PEDOT.

**
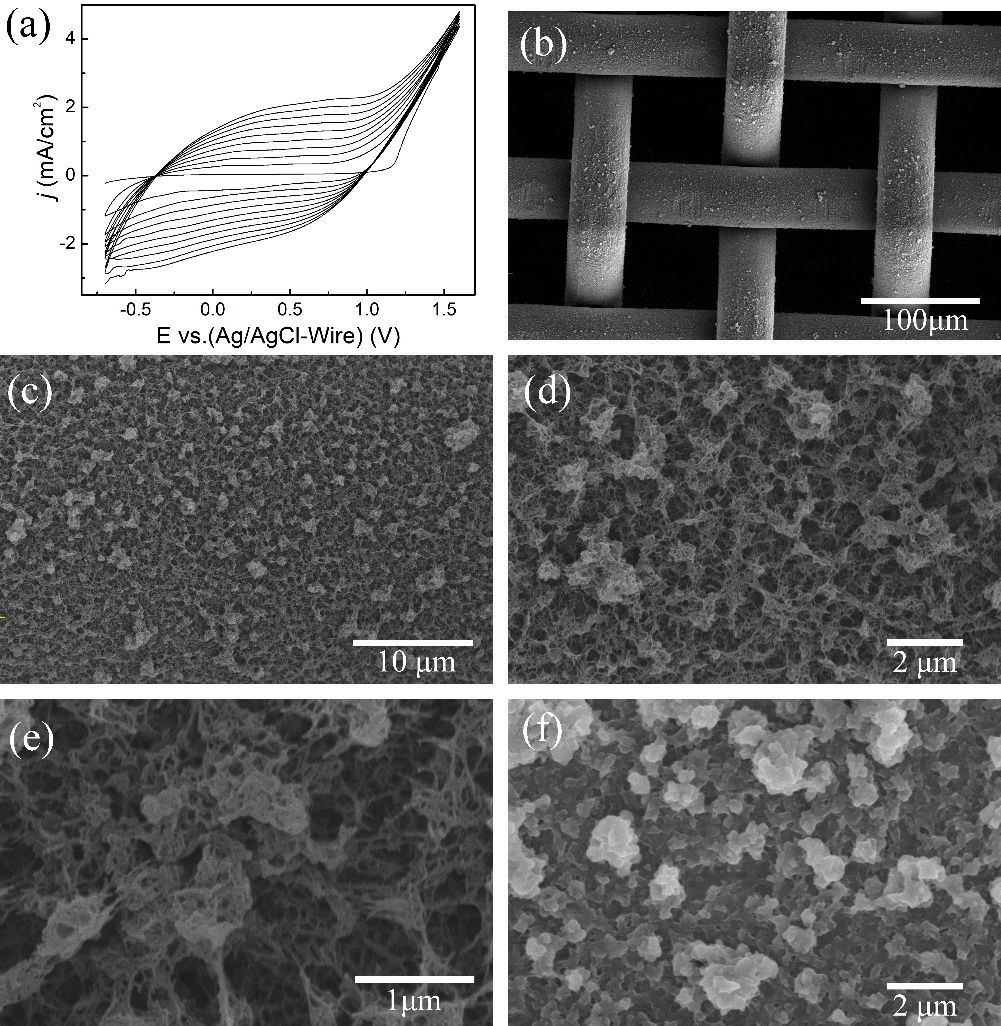
**

**Figure S1**. (a) Successive CV curves of the electrodeposition of PEDOT on the stainless steel wire mesh substrate. (b)~(e) The SEM images of the PEDOT film electrodeposited onto stainless steel wire meshes showing highly porous network-like nanostructure; (f) The SEM image of the silica-layer-encased PEDOT film (PEDOT film encapsulated in silica layer) after performing CVD of TEOS upon porous PEDOT films.

**2. Preparation of hierarchically structured porous silica coatings**

In order to obtain the hierarchically structured porous silica coatings, the as-prepared PEDOT films coating around stainless steel wires were placed in a closed desiccator (with two open vessels containing about 2 mL tetraethoxysilane (TEOS) and 2 mL aqueous ammonia solution, respectively) for the chemical vapor deposition (CVD) of TEOS. The CVD of TEOS was performed at room temperature for 48 hours (with the similar experiment conditions reported in our previous literature).^(1)^ Figure S1f was the scanning electron microscope (SEM) image of the silica-layer-encased PEDOT film (PEDOT film encapsulated in silica layer), and the structure of silica-layer-encased PEDOT films still remained porous after 48 h of CVD of TEOS. After calcinating at 500 °C for 2 hours in air to remove PEDOT templates, the hierarchically structured porous silica coatings with a morphology similar to PEDOT films were generated. The hierarchically structured porous silica coating coved all around each wire very well and have micro-/nanometer rough protrusion structure with the dimension of 0.5~2 μm (Figure 1b and c).

**3. Preparation of** **the superamphiphobic silica aerogel surface (SSAS)**

In order to generate the finer nanometer scale silica aerogel particles structure onto the hierarchically structured porous silica coatings (which have the coater micro-/nanometer scale silica structure) for minimizing further the liquid-solid contact area, a feasible soot particles templates method was performed according to the literature reported by Deng *et al*.^(2)^ The stainless-steel wire mesh covered by porous silica coatings was held upon the flame of the burning candle with 0~0.5 cm height (Scheme S2). The thin soot layers (with the thick of less than 300 nm) were deposited onto the porous silica coatings though moving the meshes with a velocity of 0.5~1 cm/s. The soot deposition is required on both sides of the meshes. The obtained sample depositing the thin soot layers exhibited light grayish (Figure S2a). As presented in Figure S2b and c, the deposited soot particles covered evenly the underlying porous silica coatings.

The deposited thin soot particles served as templates and the second CVD of TEOS was performed at room temperature for 24 hours in the similar device and experimental condition mentioned above. The obtained samples were subsequently calcinated at 500 °C for 2 hours in air to remove soot particles templates. The annealing samples were fluorinated by performing CVD of 1H,1H,2H,2H-Perfluorooctyltriethoxysilane (POTS) at room temperature for 24 hours in a closed desiccator that have two open vessels containing about 100 μL POTS and 100 μL ml aqueous ammonia solution, respectively. Thus, the superamphiphobic silica aerogel surface (SSAS) was successfully prepared. The SSAS displayed cauliflower-like multiple hierarchical structure (Figure 1c and d). In the process of preparation, the underlying porous silica coating played very roles for the SSAS to generation of the cauliflower-like multiple hierarchical structure. If the thin soot layers were deposited directly onto the stainless steel wire mesh as templates, the obtained silica aerogel surface had only a relatively flat surface (Figure S3a). Noted that excessive soot deposition could result in the generation of black soot layers on the samples surfaces, and the black soot layers would fill the pore at the bottom of the micro/nanostructure of the porous silica coatings. This is not conducive to obtain cauliflower-like multiple hierarchical structure (Figure S3b~d).

The cauliflower-like multiple hierarchical structure along with low surface energy endow the SSAS with superamphiphobicity. The superamphiphobic silica aerogel surface (SSAS) was successfully introduced the low-surface-energy fluorosilane, as evidenced by the strong F1s peak at 688.2 eV in the X-ray photoelectron spectrum (Figure S4). Figure 1c and d showed the scanning electron microscopy (SEM) image of the SSAS, and the finer length scale nanostructure particles with a size of 20 ~ 30 nm were grown uniformly onto the primary micro/nanostructure surface (Figure S5). Figure S6 showed SEM image of the cross section of superamphiphobic silica aerogel surface covering the wire, and this silica coating has a thicknes of 1~3 μm.

**
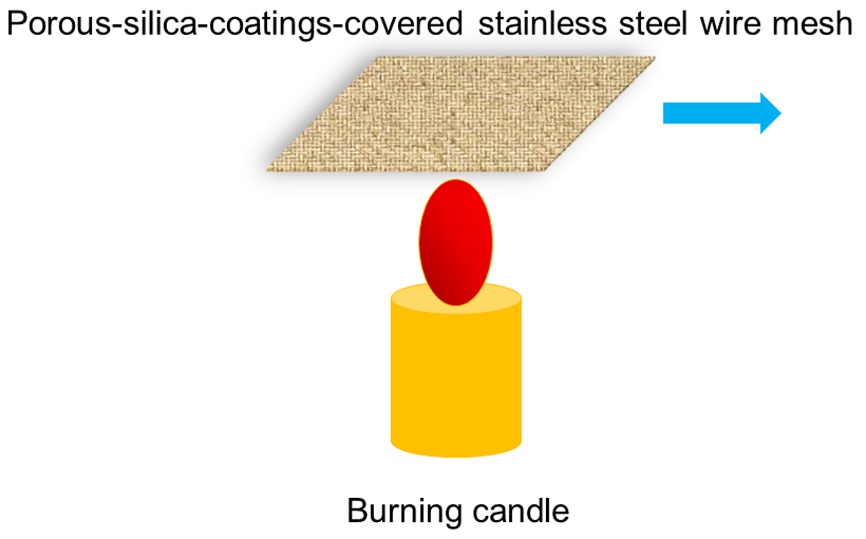
**

**Scheme S2.** The illustration of the preparation of the thin soot layers on porous-silica-coatings-covered stainless-steel wire mesh. The height between the meshes and the flame of the burning candle is of about 0~0.5 cm. The movement speed of the meshes is of about 0.5~1 cm/s.


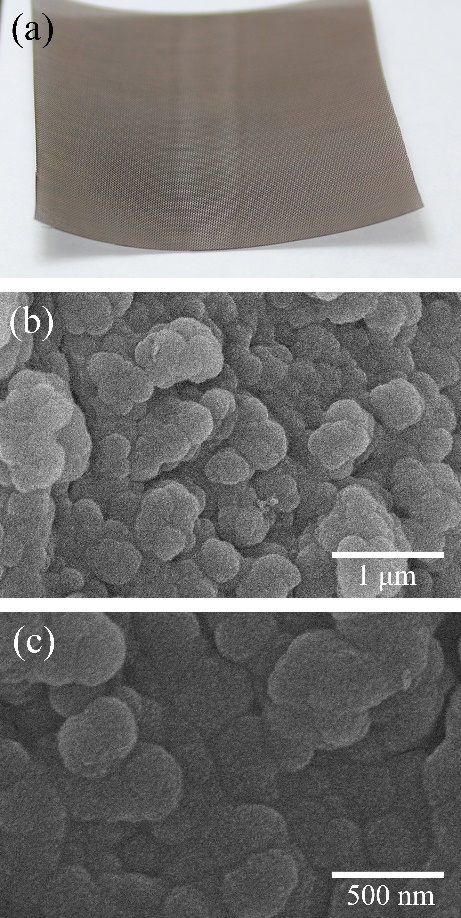


**Figure S2.** (a) The photograph of the thin soot layers deposited onto the porous-silica-coatings-covered stainless steel wire mesh, which exhibited light grayish; (b) and (c) The SEM images of the deposited soot particles layers which were covered the underlying porous silica coatings.

**
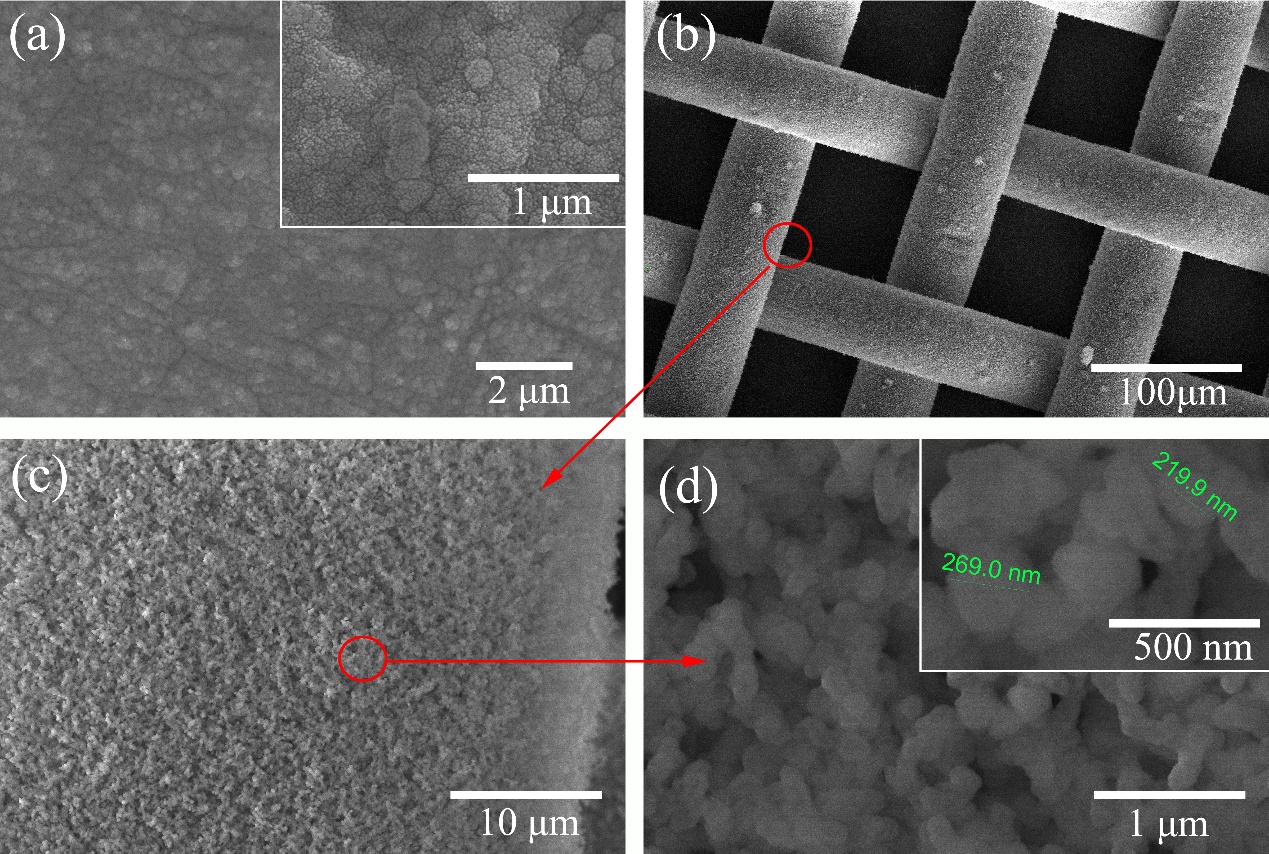
**

**Figure S3.** (a) The SEM image of the relatively flat silica aerogel surface obtained via depositing directly thin soot layers onto the stainless steel wire mesh as templates; (b)~(d) The SEM images of the silica aerogel layers on porous silica coatings obtained via depositing excessively soot particles layers (black soot layers) as templates; The pore at the bottom of the micro/nanostructure porous silica coatings were filled completely with silica particles ( with a size of 200~300 nm).


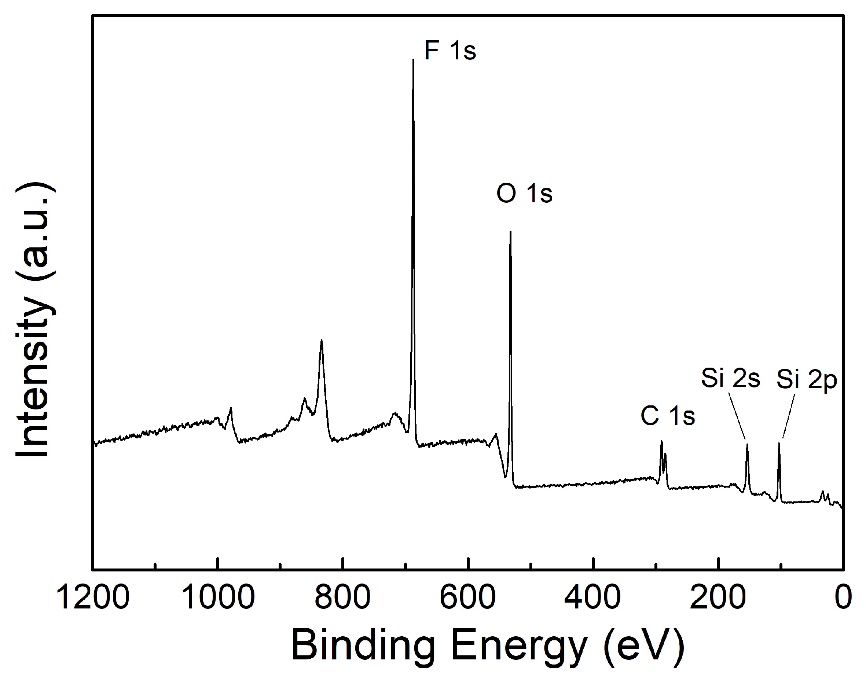


**Figure S4.**  XPS spectrum of the superamphiphobic silica aerogel surface (SSAS).


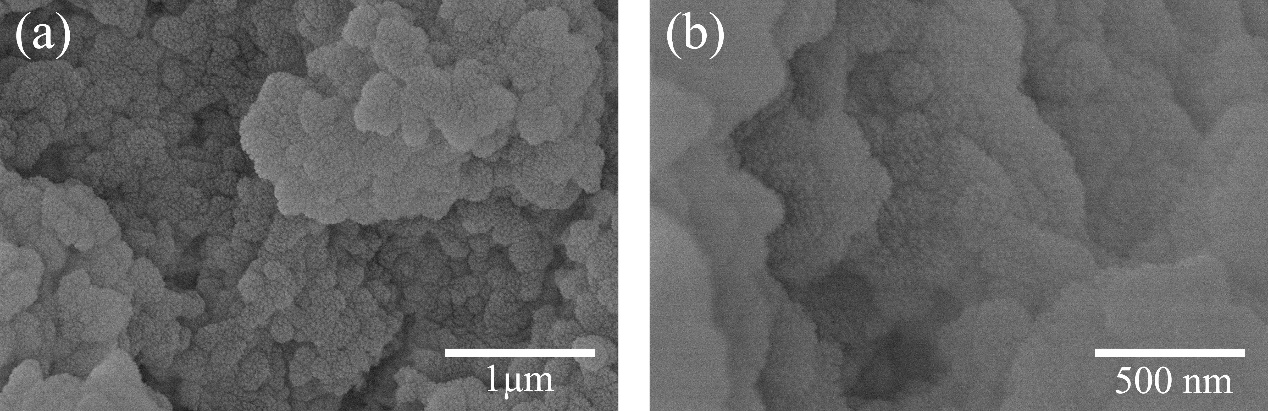


**Figure S5**. (a) SEM image of the superamphiphobic silica aerogel surface (SSAS), which exhibited the cauliflower-like multiple hierarchical nanostructure; (b) Magnified image of the superamphiphobic silica aerogel surface, and the finer nanometer scale silica aerogel particles with a size of 20 ~ 30 nm were grown uniformly onto the coarser micro/nanometer scale silica structure surface.


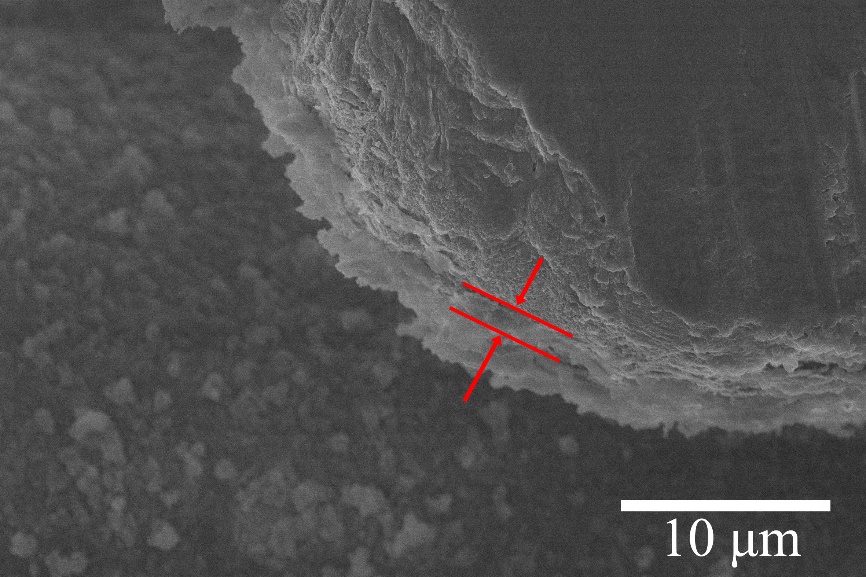


**Figure S6**. The SEM image of the cross-sectional superamphiphobic silica aerogel surface, and the thicknes of the silica coatings was of 1~3 μm.

**
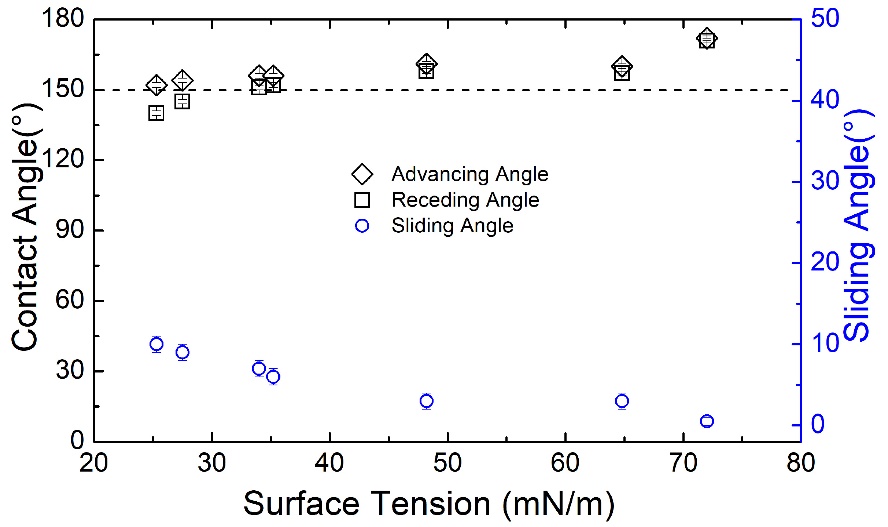
**

**Figure S7**. Advancing and receding contact angles as well as sliding angles for various surface tension liquid droplets on the superamphiphobic silica aerogel surface.

**Table S1.** Contact angle and sliding angle measurements of liquids with various surface tensions on a superamphiphobic silica aerogel surface

| Liquid | Surface tension  [mN/m] | Contact angle  [CA°] | Sliding angle  [SA °] | Advancing angle  [ *θ*_A_°] | Receding angle  [*θ*_R_ °] | Contact angle hysteresis  △*H*=*θ*_A_−*θ*_R_  [°] |
| --- | --- | --- | --- | --- | --- | --- |
| Water | 72.0 | 174° ± 0.5° | 0.5 ± 0.5° | 172 ± 0.5° | 171 ± 0.5° | 1 ± 0.5° |
| Glycerol | 64.8 | 159° ± 1° | 3 ± 1° | 160 ± 1° | 157 ± 1° | 3 ± 1° |
| Ethylene glycol | 48.2 | 160° ± 1° | 3 ± 1° | 161 ± 1° | 158 ± 1° | 3 ± 1° |
| Peanut oil | 35.2 | 157° ± 1° | 6 ± 1° | 156 ± 1° | 151 ± 1° | 5 ± 1° |
| Mineral oil | 35~31 | 156° ± 1° | 7 ± 1° | 156 ± 1° | 151 ± 1° | 5 ± 1° |
| *n*-Hexadecane | 27.5 | 153° ± 1° | 9 ± 1° | 154 ± 1° | 145 ± 1° | 9 ± 1° |
| *n*-Dodecane | 25.3 | 151° ± 1° | 10 ± 1° | 152 ± 1° | 140 ± 1° | 12 ± 1° |

**4. The** **superamphiphobicity of the fluorinated porous silica coatings (FPS)**

The porous silica coatings were fluorinated by performing CVD of POTS at room temperature for 24 hours in a similar device mentioned above (with two open vessels containing about 100 μL POTS and 100 μL ml aqueous ammonia solution, respectively). Thus, the fluorinated porous silica coatings (FPS) with superamphiphobicity was prepared, which exhibited the hierarchically structured surface possessing re-entrant curvature. Figure S8 displayed the various quasi-spherical shape droplets with different surface tension on the FPS surface. To verify the superamphiphobicity of the FPS, we measured the apparent contact angles (CA), the sliding angles (SA) and the contact angle hysteresis (△*H*) for water and various oils (Table S2). The SPS surface had contact angles (CA or *θ*) of 171°, 158°, 156°, 155°, 152° and 148° to water, glycerol, peanut oil, mineral oil, *n*-hexadecane and *n*-dodecane, respectively. The FPS surface displayed low sliding angles (SA≤10°) and low contact angle hysteresis (△*H*≤10°) for all the liquids, except *n*-dodecane with high-adhesion pinned state (with lowest surface tension).


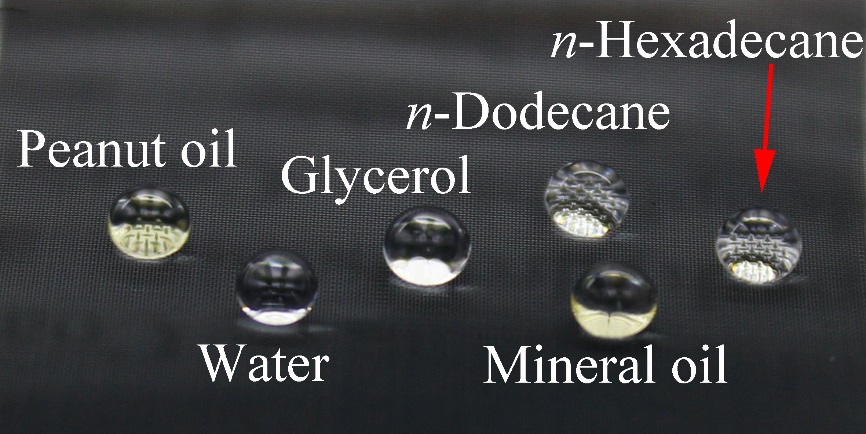


**Figure S8.** Droplets of various surface tension liquids showing the nearly spheroid shapes on the FPS surface.

**Table S2**. Contact angle (CA) and sliding angle (SA) measurements of various surface tension liquids on FPS surface.

| Liquid | CA [°] | SA [°] | Advancing angle [*θ*_A_ °] | Receding angle  [*θ*_R_°] | △*H*=*θ*_A_−*θ*_R_  [°] |
| --- | --- | --- | --- | --- | --- |
| Water | 171° ± 1° | 0.5 ± 0.5° | 171 ± 1° | 170 ± 1° | 1 ± 0.5° |
| Glycerol | 158° ± 1° | 3 ± 1° | 159 ± 1° | 156 ± 1° | 3 ± 1° |
| Peanut oil | 156° ± 1° | 7 ± 1° | 157 ± 1° | 151 ± 1° | 6 ± 1° |
| Mineral oil | 155° ± 1° | 7 ± 1° | 155 ± 1° | 149 ± 1° | 6 ± 1° |
| *n*-Hexadecane | 152° ± 1° | 10 ± 1° | 152 ± 1° | 142 ± 1° | 10 ± 1° |
| n-Dodecane | 148° ± 1° | adhesion | － | － | － |

**5.**  **Thermal stability of the SSAS mesh**

The SSAS mesh could keep the superamphiphobicity even if the mesh is upon high-temperature and  high-humidity environment in long term. The SSAS mesh with boat shape could floated on the hot water (70℃) for more than 12 hours and peanut oil droplet (~20 μL) on the SSAS mesh surface could keep free without being pinned (Figure 4b, Figure S9).


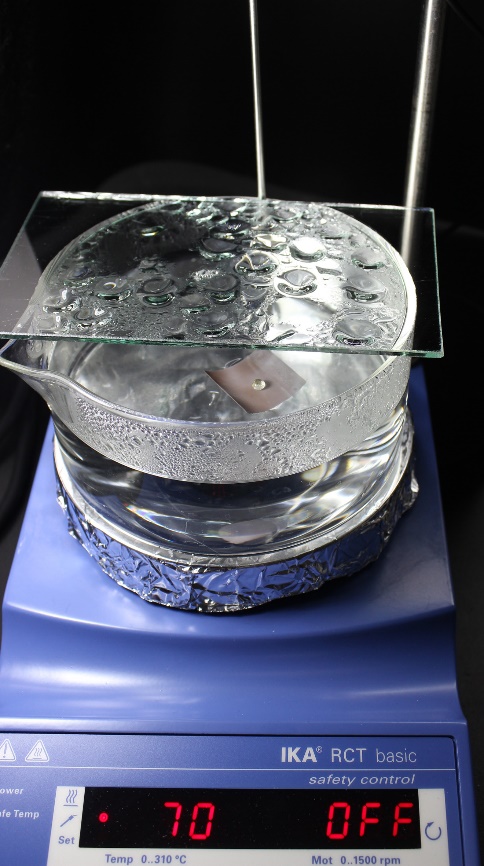


**Figure S9.** The boat made by the SSAS mesh floating on the hot water (70℃) for more than 12 hours; Peanut oil droplet (~20 μL) on the SSAS mesh surface could keep free without being pinned.

The SSAS mesh could bear the boiling water. The SSAS was immersed into boiling water for 20 minutes. Even when the SSAS mesh was under the boiling water, the reflective interfaces like a mirror was still observed (Figure S10a). After removing the SSAS mesh from the boiling water and drying naturally in air for 2 hours, The SSAS mesh remained the superamphiphobicity which was demonstrated by CA of 164° and 151° for water and peanut oil , respectively (Figure S10b and c).


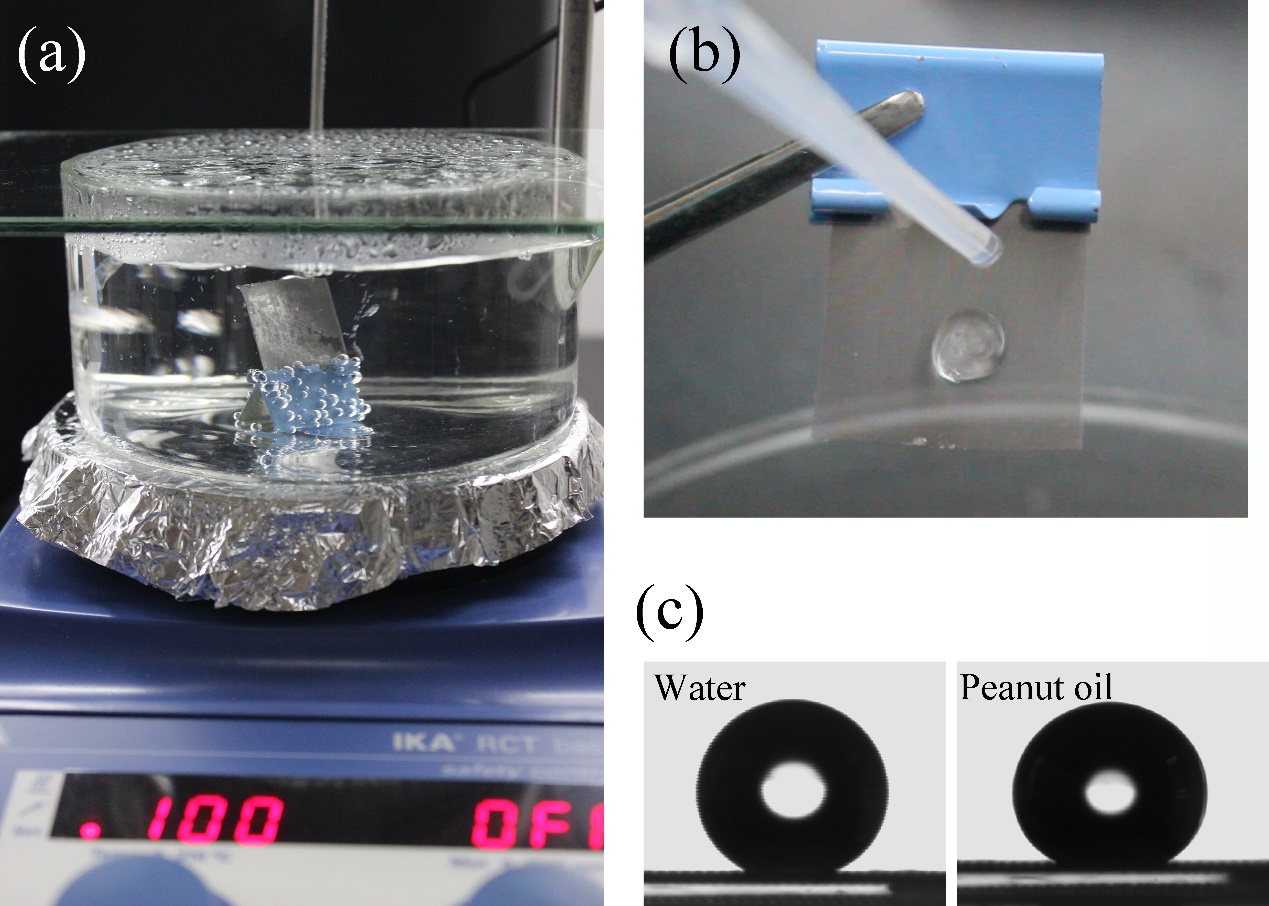


**Figure S10.** (a) Photograph of the SSAS mesh immersed into boiling water; (b) A water droplet dripping on the surface of SSAS mesh and rolling off freely; (c) The CA measurement of water and peanut oil after removing the SSAS mesh from the boiling water and drying naturally in air for 2 hours.

**Table S3.** The SSAS meshes displayed the long-team stability in a room environment and the CA and SA for water, peanut oil and *n*-hexadecane were measured after 1 and 4 months.

| **Liquid** | Water | | Peanut oil | | *n*-Hexadecane | |
| --- | --- | --- | --- | --- | --- | --- |
| **Time duration** | 1 month | 4 months | 1 month | 4 months | 1 month | 4 months |
| Contact angle [CA°] | 168° ± 1° | 168° ± 1° | 156° ± 1° | 156° ± 1° | 153° ± 1° | 152° ± 2° |
| Sliding angle [SA°] | 1 ± 1° | 1 ± 1° | 6 ± 1° | 7 ± 1° | 9 ± 1° | 10 ± 2° |


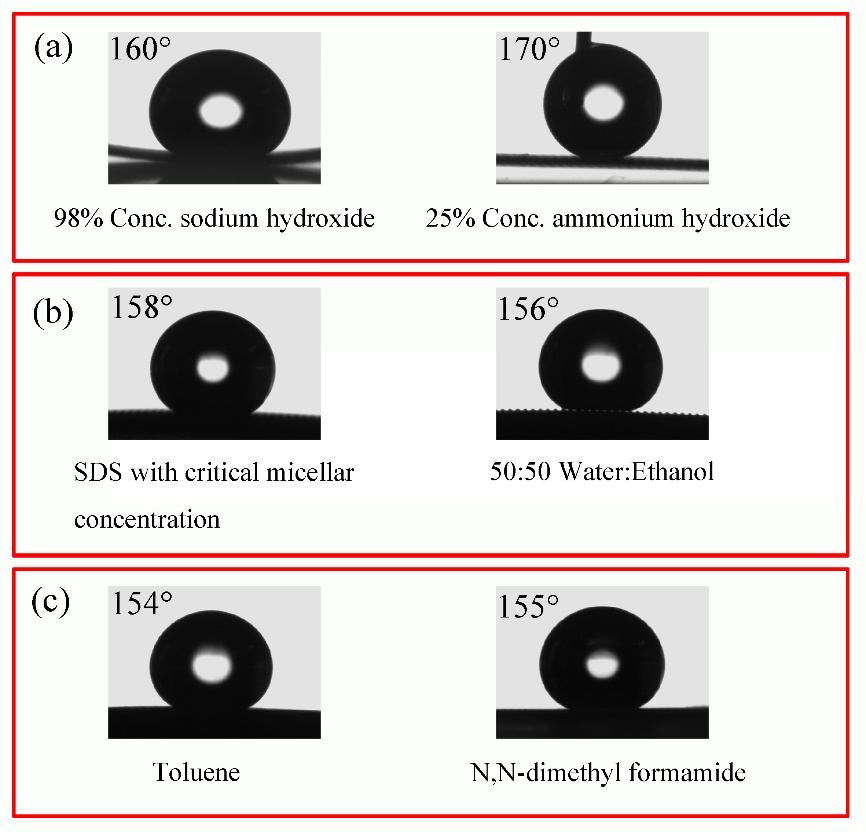


**Figure S11**. (a) The CA measurments of 98% concentrated sulfuric acid and 25% concentrated ammonia solution; (b) The CA measurments of the surfactant SDS with the critical micellar concentration and 50:50 water:ethamol; (c) The CA measurments of toluene and N,N-dimethylformamide. All the contact angles of liquids were all remained above 150°.

**6. The preparation of the small square containers.**

The SSAS mesh was made into a square container without lid (about 1 cm × 1 cm × 0.2 cm) with the preparation precess as showing in Figure S12a and b. Four side was required to avoid medium droplet rolling away during the culture (Figure S12c).

**
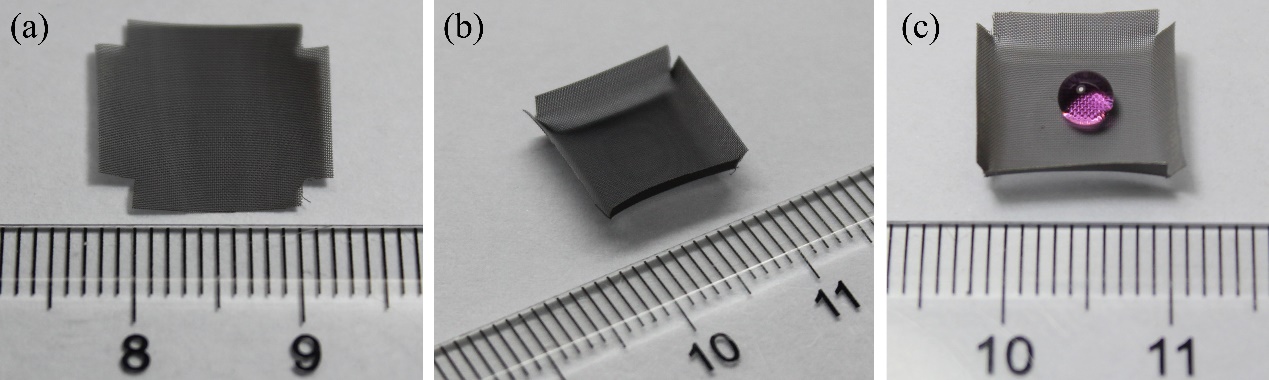
**

**Figure S12.** (a) and (b) The preparation precess of the small square containers (1 cm × 1 cm × 0.2 cm); (c) A medium droplet in the small square container.

**7. The humidity control during culture**

In this work, the cells were incubated at CO_2_ incubator with CO_2_ concentration of 5% and saturated humidity at 37 ℃ for 48 or 96h. In order to maintain a saturated humidity environment and avoid excessive evaporation of the medium droplets, a few drops of water were added around the square container at the bottom of the wells. Meanwhile, the surrounding space of the wells of 12-well plates was also filled with purified water.(Figure S13) In addition, several uncovered containers filled with purified water were placed in the incubator for creating a saturated humidity environment during culture. After 4 days incubation, the droplet containing cell spheroid could be free to roll on SSAS meth. Noted that the volume of the droplet is reduced partly due to the liquid evaporation after fluorination although we take measures to control humidity (Figure S13). The decrease of droplet volume changed the droplet curvature and apparent solid-liquid contacting area, thus promoting the formation of cell spheroid. However, excessive evaporation of the droplets could lead to the failure of the cell spheroid culture.


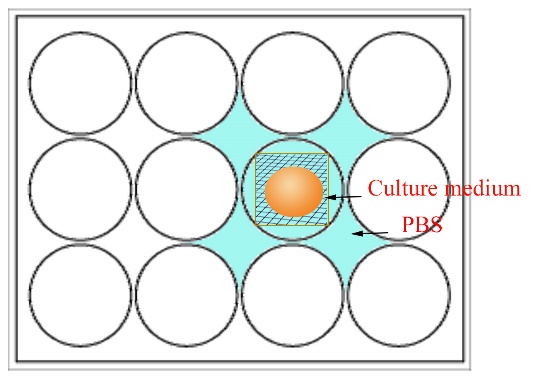


**Figure S13.** The illustration of humid conditions to prevent the evaporation of culture medium by placing the SSAS surface arround PBS.

**8. The formation of the pinned cellular spheroids on the FPS**

The FPS surface with porous micro/nanostructure morphology was also investigated on the formation of 3D cell spheroids. In contrast, the FPS surface presented the disadvantage of part cell spheroids sticking to textured substrates during 3D cellular spheroids culture with upward-culture model (up to 4 days), although the surface has the good water/oil repellence. The reason could be due to that many coater micro-/nanometer scale protuberances on the silica coating surface lacked the finer nanometer scale silica aerogel layer, leading to the stranger cell-substrates interaction. Some cells at the droplet bottom could anchor some silica protuberances during 3D cellular spheroids culture, finally resulting to the cells aggregates and the formation of the pinned cellular spheroids (Figure S14). Compared with FPS surface, the multiple hierarchical nanostructure of the SSAS surface (which was corresponding to the lower effective solid-liquid contact areas) was better to generate the completely independent cellular spheroids.


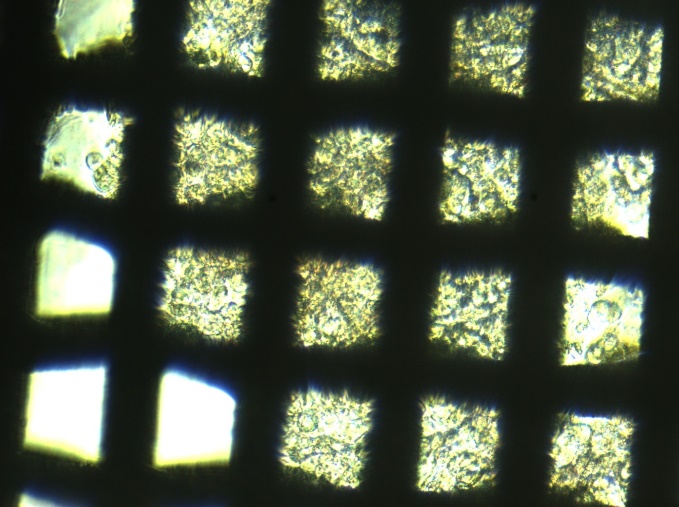


**Figure S14**. The image of the formation of the pinned cellular spheroids on the FPS.

**9. Reproducibility**

The mass production and reproducibility of spheroidal culture are quite important for real applications. To these goals, several 20 µL of culture medium droplets containing 500 to 3500 cells were placed on SSAS surface. After culture for 24h, the droplets were moved to a surface that covered fibroblast sheet to inhibit the spheroid merging. As shown in Figure S15a, cell spheroids can be prepared with uniformly spherical shape. Furthermore, these results can be reproducible for the stable antifouling properties of SSAS (Figure S15b). Herein, the SSAS exhibited stable production of 3D cell spheroids.


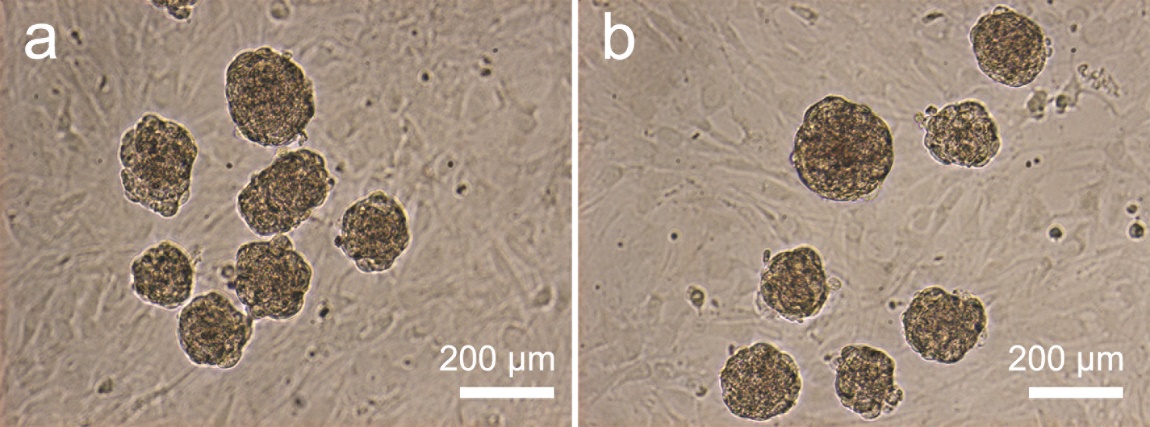


**Figure S15**. The image of 3D cell spheroids: (a) cell spheroids with different cell densities containing 500 to 3500 cells at the same time. (b) Reproducible experiments.

**10. The effect of culture medium volume on the formation of 3D cellular spheroids**

The volume of culture medium droplets could influence the formation of 3D cellular spheroids due to different apparent contacting area under its own gravity. To determine the ideal volume of culture medium droplets, the liquids with a fixed cellular concentration of 10^4^ cells/mL in volumes of 200, 100, 50 and 20 µL were dropped on SSAS meshes for 2-days culture. As shown in Figure S16, larger volume culture medium induced elliptic droplets shape and have a larger apparent flat area under its own gravity, thus leading to the formation of incompact irregulate cell aggregates. When decrease the medium volume, the droplets turned to be spherical shape with smaller apparent flat area and provided curved bottom for the formation of cell spheroids.


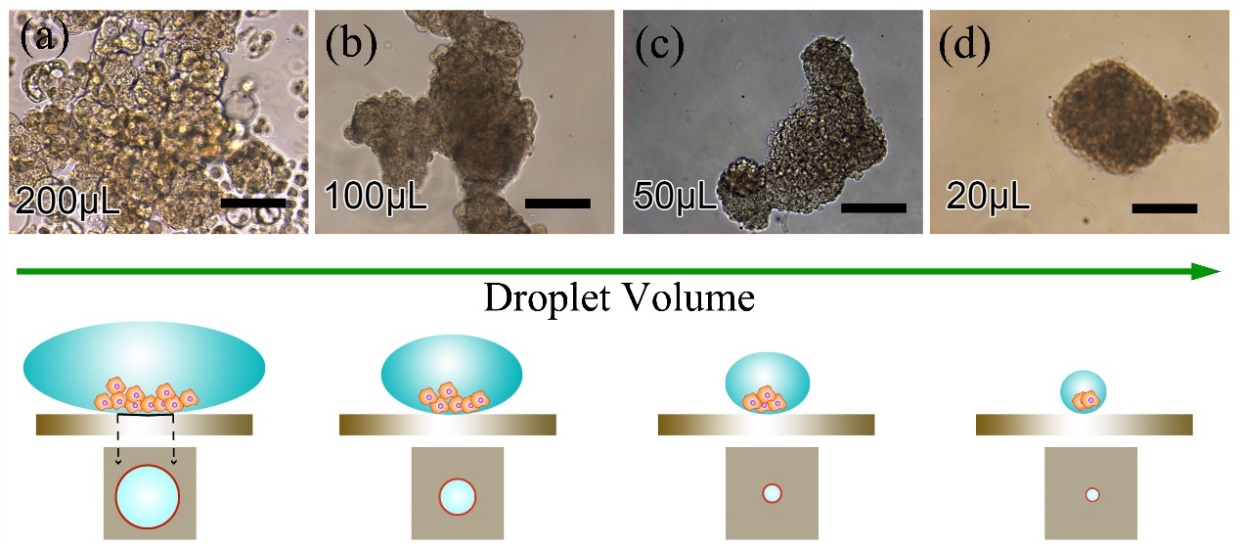


**Figure S16.** (a-d) The images (upper) and corresponding scheme (lower) of the formation of cell spheroids on the SSAS at different medium droplets volumes (200, 100, 50 and 20 μL) with a fixed cellular density of 10^4^ cells/mL, scale bar=100 μm.

**11. Viability and proliferation**

As mentioned above, the SSAS exhibited long-term antifouling properties, which was important for actual applications. What’s more, the triggering forces of spheroid formation in this method was gravity. Compared with sheer stress or hydration force, the intrinsic gravity was biosafe. Herein, it’s believed that prepared spheroids exhibited outstanding viability and proliferation. To validate this, we followed the morphologies of spheroid after culture for 96 h. As shown in Figure S17a, the diameter of prepared MCF 7 spheroid obviously increased the size due to cell growth, indicating good cell viability. Furthermore, two MCF 7 spheroids were placed together as Figure S17b. After incubation for 96h, it can be found the two individual spheroids emerged into one larger spheroid. These results fully demonstrated that SSAS provided a useful platform for cell spheroid culture and *in-situ* observation.


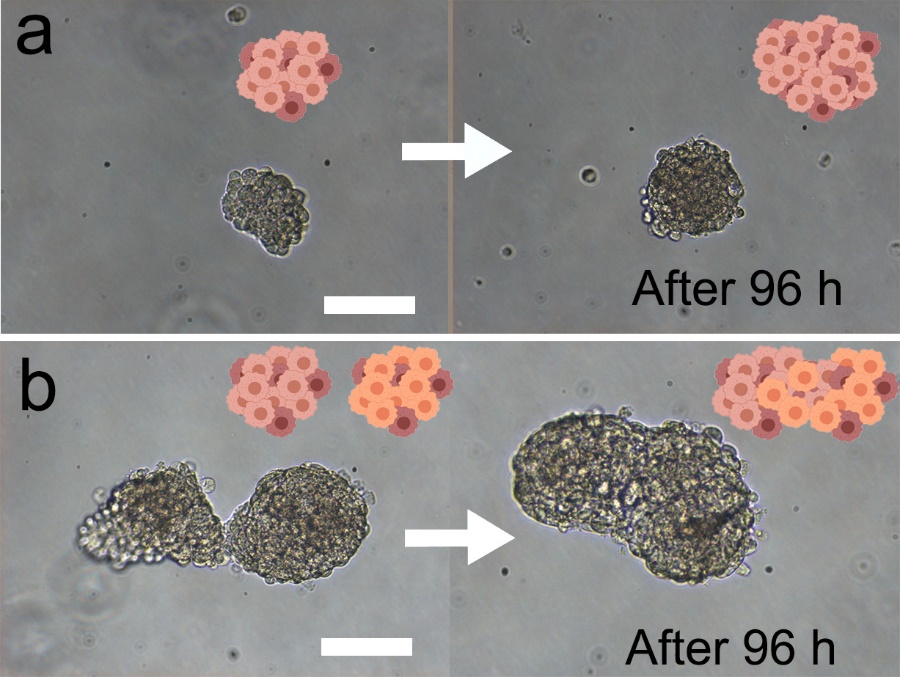


**Figure S17.** (a) The growth of spheroid after culture on SSAS surface for 96h. scale bar =150 μm. (b) The merging of two spheroids after culture on SSAS surface for 96h. scale bar=150 μm.

**12. Cell types**

To prove the versatile utility of SSAS for 3D cell spheroid culture, both cancerous cell (HeLa, C6) and non-cancerous cells Fibroblast were applied. To culture the spheroids, the as-prepared SSAS mesh was shaped into small square containers without lids (1 cm × 1 cm × 0.2 cm), each of which was transferred to an individual well of a 12-well plate. Subsequently, cell suspensions in DMEM with 10% FBS and 1% penicillin-streptomycin at a cell concentration of about 10^5^ cells/mL, was seeded into each small square container. The cells were then incubated in an atmosphere containing 5% CO_2_ at 37 °C. During the culture process, droplets of the aqueous suspension applied to the SSAS were found to be well spherical. After incubation for 24h, the spheroids were successfully prepared as Figure S18. Cancerous cell spheroids exhibited spherical shapes, which were similar with MCF-7 cell spheroids. However, only a relatively loose spheroid of fibroblasts can be obtained, which might be contributed to the slower growth speed of noncancerous cells.


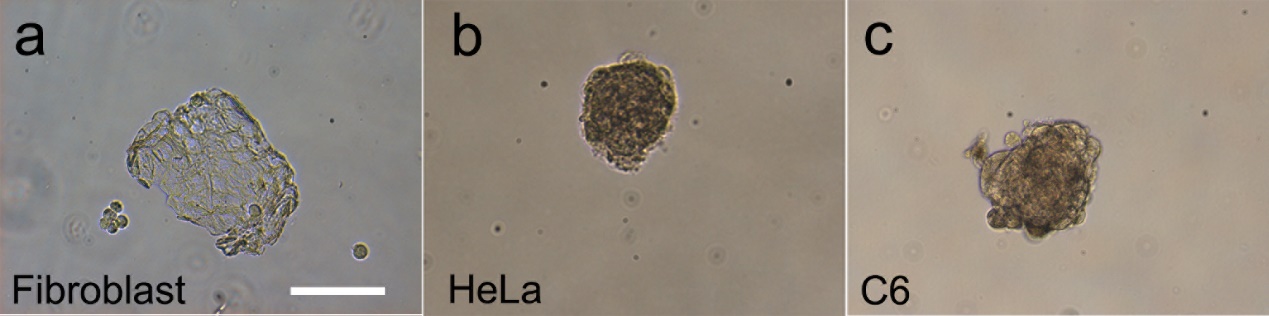


**Figure S18.** The optical images of cellular spheroids from non-cancerous fibroblast (a) and cancerous cells including HeLa (b) and C6 cells (c). scale bar =150 μm.

**13. Systematic comparison of different methods for spheroid culture**

**Table S4.** The formation time, forces, uniformity, ability for in-situ observation and long-term culture of different methods for spheroid culture.

|  | Hanging drop(3) | Hydrogel based surface(4) | | Agitation (5) | Microfludic chip(6, 7) | Floating liquid marble(8) | Magnetic NPs(9) | this work |
| --- | --- | --- | --- | --- | --- | --- | --- | --- |
| Formation time | 1 day | 7 days | | 1 day | 5-7 days | 7 days | 3-5 days | 1 day |
| Forces | Gravity +curvature | | Gravity | Shear stress | hydration force | Gravity +curvature | Magnetic Force | Gravity +curvature |
| Uniformity | Yes | NO | | NO | Yes | Yes | NO | Yes |
| In-situ Observation | NO | Yes | | NO | Yes | NO | Yes | Yes |
| Long-term culture | NO | Yes | | NO | NO | NO | NO | Yes |
| Disadvantage | Limited spheroid volume | Nonuniformity, batch-to-batch variability | | Physiological damage due to sheer force, larger amount of culture medium | Special machine  Complicated design | Laborious operation | Introduction of biotoxicity nanoparticles |  |

1. Xu, L, Chen, S, Lu, X*, et al.* Electrochemically Tunable Cell Adsorption on a Transparent and Adhesion-Switchable Superhydrophobic Polythiophene Film. *Macromol Rapid Commun*. 2015; **36**(12): 1205-10.

2. Deng, X, Mammen, L, Butt, H-J*, et al.* Candle Soot as a Template for a Transparent Robust Superamphiphobic Coating. *Science*. 2012; **335**(6): 67-73.

3. Seo, J, Lee, JS, Lee, K*, et al.* Switchable water-adhesive, superhydrophobic palladium-layered silicon nanowires potentiate the angiogenic efficacy of human stem cell spheroids. *Adv Mater*. 2014; **26**(41): 7043-50.

4. Thakuri, PS, Liu, C, Luker, GD*, et al.* Biomaterials-Based Approaches to Tumor Spheroid and Organoid Modeling. *Adv Healthc Mater*. 2018; **7**(6): e1700980.

5. Gupta, N, Liu, JR, Patel, B*, et al.* Microfluidics-based 3D cell culture models: Utility in novel drug discovery and delivery research. *Bioeng Transl Med*. 2016; **1**(1): 63-81.

6. Lee, SH, Hong, S, Song, J*, et al.* Microphysiological Analysis Platform of Pancreatic Islet beta-Cell Spheroids. *Adv Healthc Mater*. 2018; **7**(2).

7. Kwak, B, Lee, Y, Lee, J*, et al.* Mass fabrication of uniform sized 3D tumor spheroid using high-throughput microfluidic system. *J Control Release*. 2018; **275**: 201-7.

8. Vadivelu, RK, Ooi, CH, Yao, RQ*, et al.* Generation of three-dimensional multiple spheroid model of olfactory ensheathing cells using floating liquid marbles. *Sci Rep*. 2015; **5**: 15083.

9. Mattix, B, Olsen, TR, Gu, Y*, et al.* Biological magnetic cellular spheroids as building blocks for tissue engineering. *Acta Biomater*. 2014; **10**(2): 623-9.
